# *Drosophila* Fatty Acid Taste Signals through the PLC Pathway in Sugar-Sensing Neurons

**DOI:** 10.1371/journal.pgen.1003710

**Published:** 2013-09-12

**Authors:** Pavel Masek, Alex C. Keene

**Affiliations:** Department of Biology, University of Nevada, Reno, Nevada, United States of America; University of California San Francisco, United States of America

## Abstract

Taste is the primary sensory system for detecting food quality and palatability. *Drosophila* detects five distinct taste modalities that include sweet, bitter, salt, water, and the taste of carbonation. Of these, sweet-sensing neurons appear to have utility for the detection of nutritionally rich food while bitter-sensing neurons signal toxicity and confer repulsion. Growing evidence in mammals suggests that taste for fatty acids (FAs) signals the presence of dietary lipids and promotes feeding. While flies appear to be attracted to fatty acids, the neural basis for fatty acid detection and attraction are unclear. Here, we demonstrate that a range of FAs are detected by the fly gustatory system and elicit a robust feeding response. Flies lacking olfactory organs respond robustly to FAs, confirming that FA attraction is mediated through the gustatory system. Furthermore, flies detect FAs independent of pH, suggesting the molecular basis for FA taste is not due to acidity. We show that low and medium concentrations of FAs serve as an appetitive signal and they are detected exclusively through the same subset of neurons that sense appetitive sweet substances, including most sugars. In mammals, taste perception of sweet and bitter substances is dependent on phospholipase C (PLC) signaling in specialized taste buds. We find that flies mutant for *norpA*, a *Drosophila* ortholog of PLC, fail to respond to FAs. Intriguingly, *norpA* mutants respond normally to other tastants, including sucrose and yeast. The defect of *norpA* mutants can be rescued by selectively restoring *norpA* expression in sweet-sensing neurons, corroborating that FAs signal through sweet-sensing neurons, and suggesting PLC signaling in the gustatory system is specifically involved in FA taste. Taken together, these findings reveal that PLC function in *Drosophila* sweet-sensing neurons is a conserved molecular signaling pathway that confers attraction to fatty acids.

## Author Summary

The gustatory system is largely responsible for interpreting the nutritional value and potential toxicity of food compounds prior to ingestion. The receptors and neural circuits mediating the detection of sweet and bitter compounds have been identified in fruit fly, but neural mechanisms underlying detection of other taste modalities remain unclear. Here, we demonstrate through multiple lines of inquiry that fatty acids represent an appetitive cue that is sensed through the primary gustatory system. We find that fatty acids are detected by the same neurons that are also sensitive to sugars. Remarkably, the phospholipase C pathway, which mediates gustatory perception in mammals, is required in *Drosophila* for the taste of fatty acids but not sugars or bitter substances. Our findings reveal, for the first time, that fruit flies are capable of fatty acid taste, and identify a conserved molecular signaling pathway that is required for fatty acid feeding attraction.

## Introduction

The gustatory system is critical for interpreting the nutritional value and potential toxicity of food compounds prior to ingestion. Nutritionally relevant food components are detected through specialized taste receptors expressed in sensory neurons that are broadly tuned to specific taste modalities [1]–[3]. Flies are also capable of detecting the caloric content of sugars through satiation feedback from internal sensors [4]–[7]. Taste represents an analytic sense, and unlike olfaction or vision, distinct taste modalities are sensed and processed independently from each other. The *Drosophila* gustatory system is divided into two main functional pathways that either detect appetitive sugars or aversive bitter substances [1], [8], [9]. Of the five basic taste qualities described in humans - sweet, sour, salty, umami, and bitter, fruit flies have been shown to detect tastants encompassed by only three of these taste modalities - sugars, bitter and salt [7], [10], [11]. Foods containing sugars, dietary lipids, and amino acids represent significant energy sources, and their presence tends to be attractive and promote consumption. In mammals, dietary lipids signal through mechanosensory and olfactory neurons, as well as postingestive feedback [12]–[15]. Dietary lipids are comprised of both triacylglycerides and fatty acids (FAs), and growing evidence suggests that it is the free fatty acids that are detected by the gustatory system [16]–[23]. Fat represents a potent food source that yields more than twice the amount of energy as sugars per unit of mass. An understanding of how dietary FAs are sensed will provide critical insight into feeding choice and gustatory processing.

While much is known about the detection and processing of sweet and bitter tastants in *Drosophila*, the neural basis for fat taste is unclear. *Drosophila* detect short-chain saturated FAs in free walking paradigms and they prefer low, while avoiding high FA concentrations [24]. Here we show that detection of a variety of FAs by the fly gustatory system induces a robust feeding response. These FAs serve as a dietary supplement with a potency that is comparable to sugars. FAs are perceived as appetitive at low and medium concentrations, and aversive at high concentrations. FA perception is independent of the olfactory system and acidity and instead requires the same gustatory sensory neurons that detect sugars. In mammals, phospholipase C (PLC) signaling is a critical second messenger required for taste. Our results demonstrate that PLC is uniquely required to sense FAs in *Drosophila*, revealing a conserved gustatory pathway that is independent from that required for sugar signaling.

## Results

**Figure 1:**
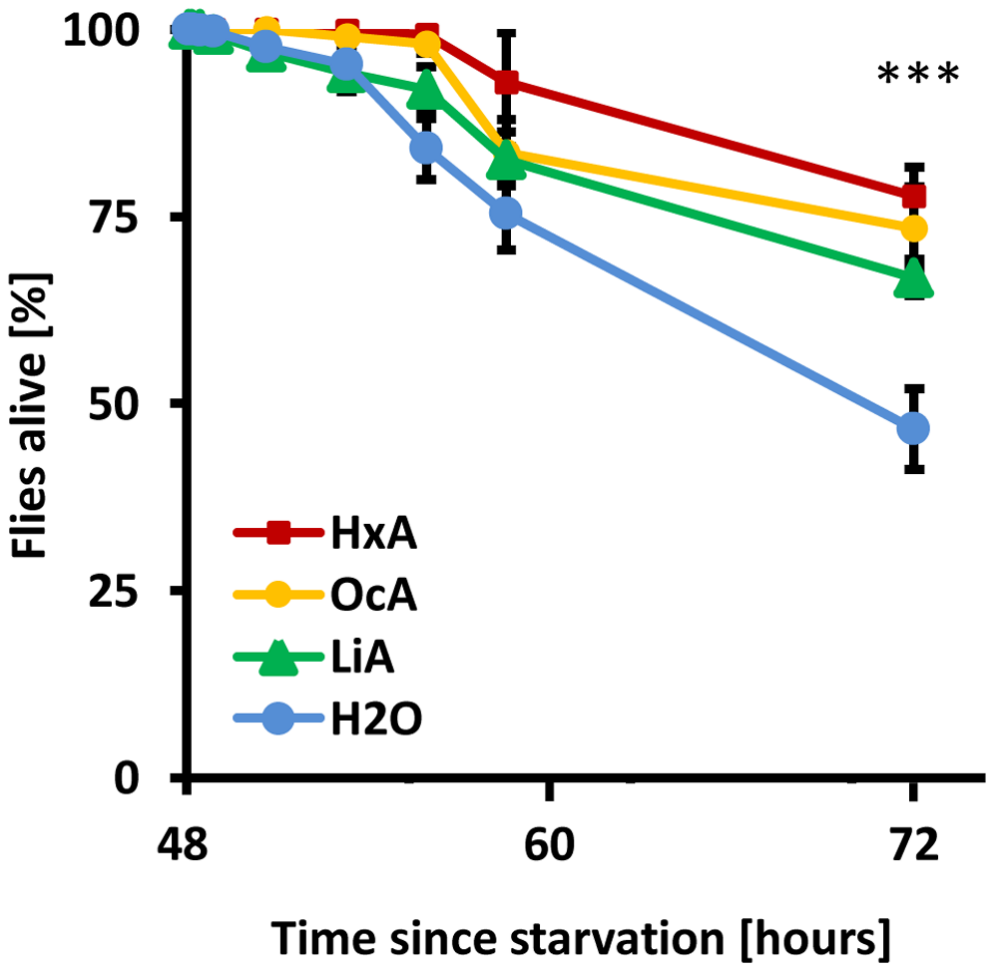
Dietary fatty acids are sufficient for survival. Flies were starved for 48% HxA, OcA and LiA. All three fatty acids alone sufficient for higher survival compared to water alone. All data, mean ± s.e.m. *** p<0.001 compared to water control.

To determine whether dietary fatty acids are sufficient for survival, flies were fed a diet composed exclusively of FAs (Hexanoic acid – HxA, Octanoic acid – OcA, or Linoleic acid – LiA). HxA and OcA are short-chain saturated FAs that are naturally found in animal and plant products, including goat milk and coconut oil, and that are in the diet of some *Drosophila* species [24]. LiA is a long-chain unsaturated FA that is essential for human diet. The feeding preference and survival on FA diet was measured in a capillary feeding assay (CAFE). Approximately 30–60 wild-type flies were starved for 48 hours prior to being placed in a vial with two capillary tubes: one containing 1% solution of various FA, and the other water. The number of surviving flies was measured over the course of 24 hours. Flies fed on FAs had a higher survival rate after 24 hours than control flies feeding on water alone (P<0.01 for all concentrations, ANOVA; Fig. 1). A dose-response curve revealed that low concentrations of HxA prolong survival in previously starved *Drosophila*. Flies were offered 1%, 0.4% or 0.1% solution of HxA and the numbers of surviving flies were measured over the course of 24 hours (Fig. S1). Flies fed 1% HxA showed no lethality throughout the length of the experiment. Flies fed 0.4% and 0.1% HxA showed a progressively decreasing survival rate that negatively correlated with concentration. For all concentrations of HxA tested, flies survived longer than control flies feeding on water alone (P<0.01 for all concentrations). Taken together, these findings suggest that dietary FAs are metabolizable and partially sufficient for survival.

**Figure 2:**
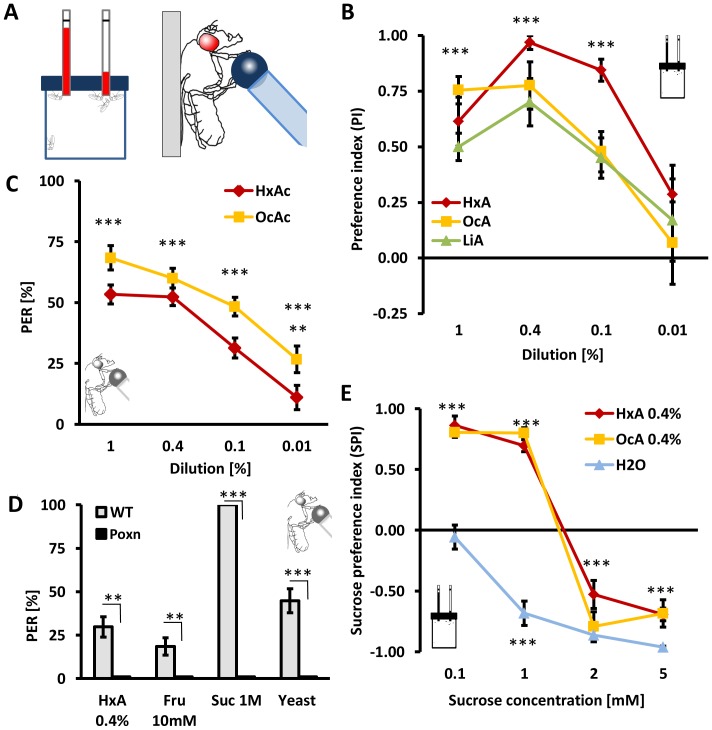
Fatty acids are appetitive tastants. A) Left: Capillary feeding assay CAFE provides flies a choice between a nutrient and control tube. Right: In Proboscis extension reflex (PER) assay, flies are stimulated with a tastant on their feet and they respond with extension of their proboscis to appetitive substances in attempt to feed. The probability of their response is proportional to the level of starvation and the hedonic value of the substance. B) HxA, OcA, LiA acids are preferred over water in CAFE at concentrations ranging from 1–0.1%. C) The PER elicited in response to presentation of HxA or OcA. Concentrations of 1% to 0.01% elicit significant PER responses that are concentration dependent. D) *Poxn* mutants lack all peripheral taste neurons and show no response to HxA, fructose, sucrose or yeast. E) Appetitive response to FAs in two-choice feeding assay is comparable to low concentrations of sugars and is concentration dependent. Intake of 0.4% HxA and 0.4% OcA were measured against different concentrations of sucrose. Flies prefer sucrose to water at concentrations of 0.5 mM or greater but prefer 0.4% HxA and 0.4% OcA to 1 mM sucrose or lower, while sucrose is preferred at concentrations of 2 mM and greater (p<0.001). All data, mean ± s.e.m. ** p<0.01, *** p<0.001; NS, not significant, t-test.

When provided a choice in the CAFE assay (Fig. 2A) between FAs and water, flies strongly preferred FAs (HxA, OcA, and LiA) at concentrations of 0.1% or greater (P<0.001 for all groups; Fig. 2B). Additionally, we found that flies display robust preference for oleic (mono-unsaturated, omega-9), decanoic and myristic acids (both saturated FAs) at concentrations of 0.4% in the CAFE assay (data not shown).

Dietary sugars are detected through gustatory receptors on the tarsi and proboscis as well as through internal metabolic sensors [5], [6], [25], [26]. To investigate whether flies detect fatty acids through the peripheral gustatory system or through internal nutrient sensors, we measured the reflexive feeding response in Proboscis Extension Reflex (PER) assay (Fig. 2A). Briefly, a small volume of either OcA or HxA was applied to the fly tarsi, and PER was measured as previously described [27], [28]. When measuring PER, the tastant does not touch the proboscis, and therefore, cannot be ingested. Presentation of HxA or OcA dilutions ranging from 1% - 0.01% resulted in robust PER that was significantly greater than the response to water (P<0.001 for all groups, except P<0.01 for 0.01% HxA), suggesting that peripheral gustatory receptors are sufficient for detection of FAs (Fig. 2C). In *Poxn* mutant flies, external chemosensory sensillae are converted to mechanosensory sensillae [29]. These mutants can detect nutrients through internal sugar receptors, but do not display gustatory responses to tastants [6]. The PER response to 0.4% HxA (as well as to sugars and yeast) was abolished in *Poxn* mutant flies, further indicating that FAs are detected through peripheral sensory receptors (Fig. 2D).

The dietary sugars sucrose and fructose are strong gustatory attractants [30]. We sought to determine if flies can distinguish between FAs and sugars by testing whether flies exhibit concentration-dependent FA/sugar preference. To determine the sucrose response threshold, flies were provided a choice between water and sucrose in concentrations ranging from 0.1 to 5 mM in the CAFE assay and total ingestion was measured. Flies displayed strong preference for sucrose at 0.5 mM and higher (P<0.001 for all groups) (Fig. 2E). When offered a choice between 0.4% HxA, or OcA, and a range of sucrose concentrations, flies preferred FAs over sucrose at concentrations less than 1 mM (P<0.001 for sucrose 0.1 mM and 1 mM), while sucrose was preferred at concentrations greater than 2 mM (P<0.001 for all groups for sucrose at 2 mM and 5 mM; Fig. 2E). These results reveal that flies display a concentration-dependent preference for FAs over sucrose. To determine whether concentration-dependent FA/sugar choice is specific to sucrose, we measured feeding preference comparing 0.4% HxA to a range of fructose concentrations. We found that flies similarly preferred HxA over fructose concentrations less than 1 mM (P<0.001 for fructose 0.5 mM and 1 mM) and fructose at concentrations greater than 2 mM (P<0.001 for fructose 2 mM and 5 mM) (Fig. S2). Taken together, these findings reveal that at certain concentrations, flies prefer FAs over sugars as a food source.

**Figure 3:**
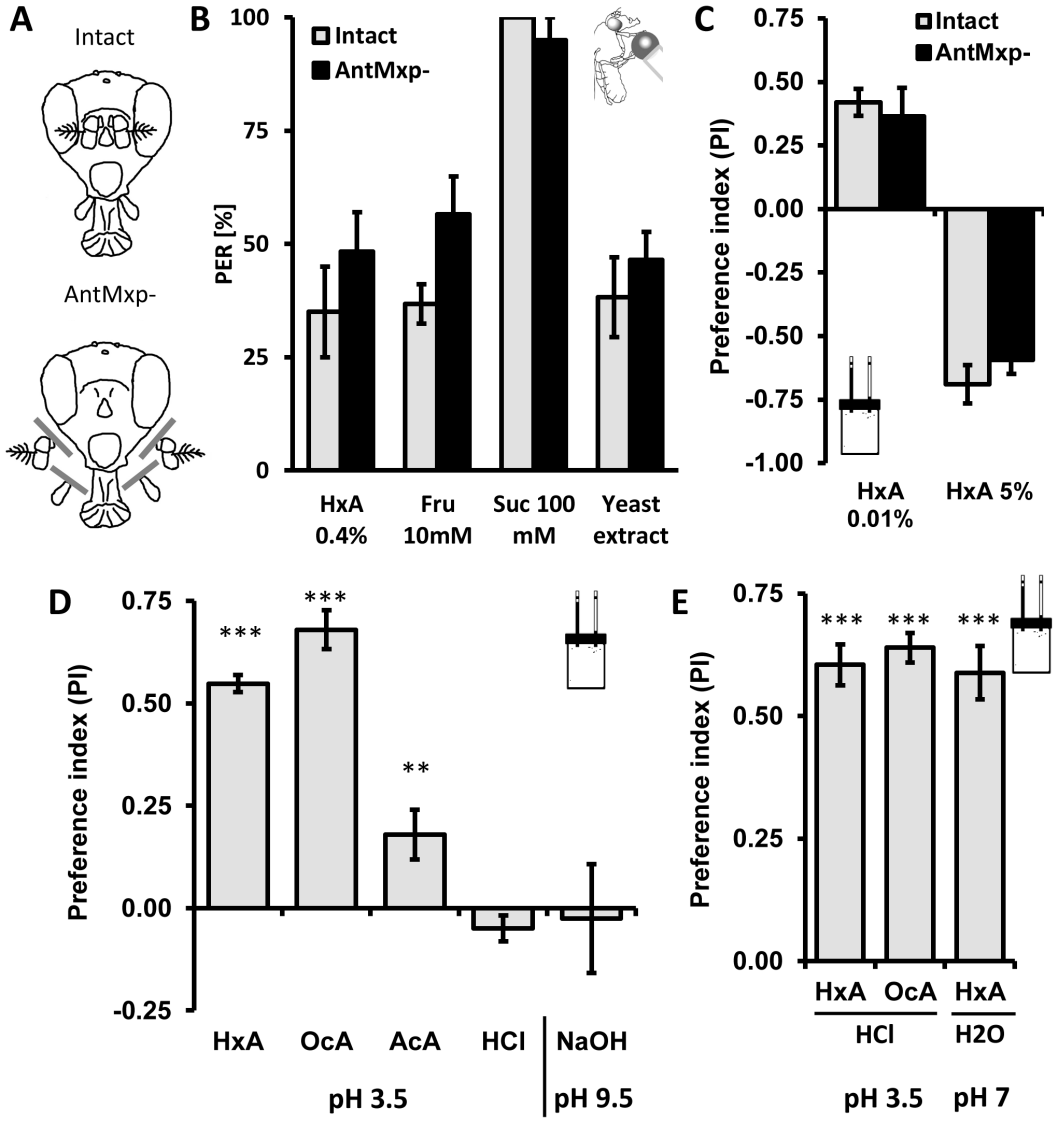
The primary olfactory system and acidity are dispensable for perception of fatty acids. A) Flies were split in two groups, one used as intact control (intact) and the other with antennae and maxillary palps surgically removed (antmxp-). B) No differences were observed in flies with antennae and maxillary palps surgically removed (antmxp-) compared to intact controls (intact) while flies were tested for PER response to HxA, fructose, sucrose and to yeast extract. C) Intact flies and antmxp- flies do not show difference in preference for FA (HxA 0.01%) or in avoidance (HxA 5%) in the CAFE assay. D) Preference of FAs is specific and is pH independent. Flies strongly prefer 0.1% HxA and 0.1% OcA, show weak preference for 0.1% acetic acid (AcA) and no preference for 0.01% hydrochloric acid (HCl) when tested at pH∼3.5. Flies do not show preference for high pH (NaOH, pH∼9.5). E) Flies prefer HxA and OcA over HCl at matched acidity (pH∼3.5). HxA diluted in PBS buffer tested against PBS retains strong preference for HxA at the neutral pH (pH∼7.2–7.4). All data, mean ± s.e.m. ** p<0.01, *** p<0.001; NS, not significant, t-test.

Flies detect food through olfactory neuron dendrites that localize to the antennae and maxillary palps, and through gustatory neurons in the proboscis and legs [31]–[33]. These chemosensory organs are located relatively close to each other and are used for multimodal sensory processing of food cues [34]. To determine whether detection of FAs occurs independently from the primary olfactory system, we surgically removed antennae and maxillary palps, generating anosmic flies that lack olfactory organs [34], [35] (Fig. 3A). No significant differences were observed in the PER response to HxA, sugars (fructose and sucrose) or yeast extract between intact flies and flies lacking olfactory organs (AntMxp-; P>0.05, t-test for each pair; Fig. 3B). Preference for low concentration of HxA (0.01%) and avoidance of a high concentration of HxA (5%) in the CAFE assay did not differ between anosmic and intact flies (0.01% HxA P>0.568, 5% HxA P>0.406), suggesting olfaction is not required for HxA feeding preference or avoidance (Fig. 3C). Taken together, these findings indicate that FA attraction is independent of the primary olfactory system.

Fruit flies can sense acids and we sought to determine whether gustatory FA detection is dependent on acidity [36]. We tested preference for 1% HxA and OcA, as well as 0.1% acetic acid and 0.01% HCl (pH∼3.5–3.6 for all) in the CAFE assay. We also measured preference for the base NaOH (pH∼9.5) to determine if high pH affects preference. Flies strongly preferred both HxA and OcA to water (P<0.001 for both groups). Flies also preferred acetic acid (P<0.008) but the preference was significantly lower than preference to FAs (Fig. 3D, P<0.01 for both FAs). No significant preferences were observed with HCl (P>0.094) or NaOH (P>0.660; Fig. 3D) suggesting that flies are generally not attracted to acidic or basic substances (Fig. 3D). We tested the same concentrations of HxA and OcA against HCl in the CAFE assay. Despite matching pH, flies robustly preferred HxA and OcA over HCl (P<0.001 to both FAs), suggesting that FA taste is mediated through chemical structure rather than low pH (Fig. 3E). To directly measure whether acidity is required for FA taste, we adjusted the pH of 0.1% HxA to neutral (pH∼7–7.2) by adding PBS buffer (pH 7.4). Flies strongly preferred pH-neutral HxA to PBS, confirming that FA taste is independent of acidity (P<0.001; Fig. 3E).

**Figure 4:**
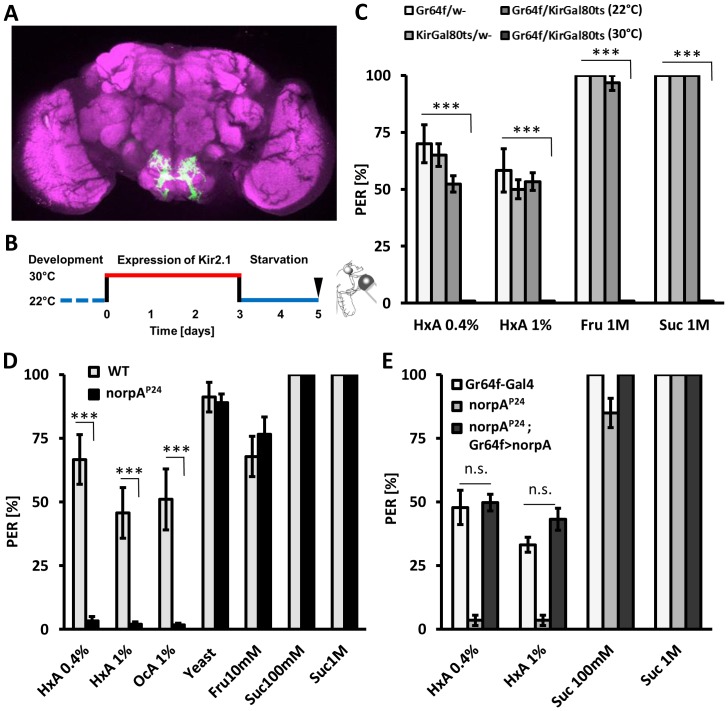
Fatty acids taste requires intact PLC signaling specifically in sugar-sensing neurons. A) Expression of GFP under the control of Gr64f-GAL4 (green). Neuropile regions are labeled by nc82 antibody (magenta). The sweet-sensing neurons ramify throughout the suboesophageal ganglion. B) Specific neurons are silenced by expression of Kir2.1-GAL80^ts^ at 30°C during adulthood. C) PER response to HxA is abolished with adult-specific silencing of sugar-sensing neurons (Gr64f). Flies with silenced Gr64f neurons (Gr64f-GAL4>Kir2.1-GAL80^ts^ at 30°C) showed significantly reduced PER compared to control flies harboring either UAS-Kir2.1;GAL80ts or Gr64f-GAL4 alone or to flies with not activated Kir2.1 (Gr64f-GAL4>Kir2.1GAL80^ts^ at 22°C) (p<0.001). D) *norpA* ^P24^ mutant flies are deficient in sensing HxA but respond normally to water and other tastants including yeast, fructose, and sucrose. E) Restoring *norpA^P24^* function selectively in Gr64f neurons by expressing the norpA transgene under control of Gr64f-GAL4 (Gr64f-GAL4>UAS-*norpA*) rescues PER response to HxA compared to mutant control (*norpA* ^P24^;+) (p<0.001) to the level of control carrying intact *norpA* allele (Gr64f-GAL4/+) (p>0.05). All data, mean ± s.e.m. *** p<0.001; NS, not significant, t-test.

Flies sense sugars through gustatory receptor neurons that express *gustatory receptor* 64f (Gr64f) and can be labeled with Gr64f-GAL4 (Fig. 4A), and aversive tastants through bitter-sensing neurons labeled by Gr66a-GAL4 [8], [37], [38]. These complementary populations of gustatory neurons can be selectively silenced through expression of the inward rectifying K+ channel Kir2.1 [39]. We expressed Kir2.1 under control of Gr64f-GAL4 to determine whether sweet-sensing neurons also detect FAs. To avoid potential developmental defects caused by silencing neurons throughout development, Kir2.1 expression was limited to adulthood with GAL80^ts^ [40], [41]. Briefly, adult-specific Kir2.1 expression was induced in sweet-sensing neurons by incubating 3 day-old flies at the non-permissive temperature of 30°C for 72 hours prior to testing. Flies were then starved for 48 hours at 22°C. Flies expressing Kir2.1 in sweet-sensing neurons and control flies were all tested at 22°C to prevent confounds of testing temperature on feeding behavior (Fig. 4B). Silencing sugar-sensing neurons (Gr64f-GAL4>UAS-Kir2.1,GAL80^ts^) abolished PER response to fructose and sucrose while control flies displayed robust PER (P<0.001 compared to all controls, Fig. 4C). Strikingly, silencing Gr64f neurons also abolished PER response to all tested concentrations of HxA (P<0.001 compared to all controls), indicating that Gr64f-expressing neurons are also required for HxA sensing (Fig. 4C). Control flies of the same genotype (Gr64f-GAL4>UAS-Kir2.1,GAL80^ts^) maintained at 22°C do not express Kir2.1, and PER response to sugars or HxA was normal (p>0.05 compared to other control groups, p<0.001 to the same genotype at 30°C). These findings indicate that FAs are sensed by, and confer feeding through, the same population of gustatory neurons that detect sugars.

In vertebrates, the tastes of sweet, bitter, and amino acids are dependent upon phospholipase C (PLC) signaling [42]–[44]. We measured PER in response to FAs in flies mutant for *no receptor potential A* (*norpA*), a fly ortholog of mammalian PLC. The mutant *norpA* ^P24^ is a null allele and has previously been reported to have deficits in visual performance [45]. *norpA* ^P24^ flies displayed dramatically reduced PER in response to HxA and OcA compared to wild-type controls (P<0.001 for both groups), suggesting that *norpA* is required for FA taste (Fig. 4D). However, PER response to fructose, sucrose, and yeast were comparable in *norpA* ^P24^ and control flies (P>0.05 for all groups), suggesting that *norpA* activity is required for sensing FAs specifically (Fig. 4D). To localize the neurons where *norpA* is required for FA taste, we selectively restored *norpA* function to the sweet-sensing neurons. Flies with *norpA* expression limited to the Gr64f-expressing neurons showed greater PER response to HxA than *norpA* ^P24^ mutants (P<0.001 for both HxA concentrations) and were statistically indistinguishable from control flies (Gr64f-GAL4, 0.4% HxA P = 0.808 and 1% HxA P = 0.082). These findings suggest that *norpA* functions in sweet-sensing neurons to detect FAs (Fig. 4E). No rescue was observed in flies with *norpA* expression limited to the *rhodopsin-1* expressing neurons, where *norpA* is required for proper function of a visual system or in bitter-sensing Gr66a-expressing neurons (Fig. S3), confirming that the rescue of *norpA* in sweet- sensing neurons is not due to leakiness of the rescue transgene. To confirm rescue results, *norpA* was selectively targeted in sweet-sensing neurons through expression of two-independent RNAi lines. Transgenic flies with Gr64f-GAL4 and *norpA*-IR1 or *norpA*-IR2 displayed significantly reduced PER to HxA compared to control flies harboring Gr64f-GAL4 or UAS-RNAi transgenes alone (Fig. S4; P<0.01), confirming that *norpA* is required in sweet-sensing neurons for FA taste. Both sucrose and fructose response of flies with RNAi-*norpA* expressed under control of Gr64f-GAL4 was comparable to controls confirming that *norpA* expression in sweet-sensing neurons is selectively required for FA sensing. The receptors TRPM5 and TRPA1 signal through the PLC gustatory pathway in mammals and are proposed to be a polyunsaturated FA sensor in *Drosophila* and mammals [46], [47]. In *Drosophila*, TRPA1 is also expressed in bitter-tasting neurons and confers avoidance of electrophiles [48], [49]. However, TRPA1 mutant flies (dTrpA1^ins^) display a wild-type response to FAs suggesting TRPA1 is dispensable for FA taste in *Drosophila* (Fig. S3) [50]. We conclude that FA taste in flies requires *norpA*/PLC function in sweet-sensing neurons, indicating that fly FA taste utilizes a pathway conserved in mammals.

## Discussion

Our findings demonstrate that *Drosophila* display robust attraction and feeding response when presented with FAs. This preference is specific to the gustatory properties of FAs and is independent from acidity and smell. The response to FAs is mediated by a small population of neurons in the gustatory system that is also responsible for perception of sugars and glycerol [8], [51]. Functional *norpA*/PLC signaling in these neurons is required for FA-induced feeding response, but is dispensable for sugar sensing, suggesting that distinct signaling pathways mediate sugar and FA response in these cells. Therefore, these findings have important implications for understanding how animals detect, and are attracted to, fatty acids.

### Fatty acids are detected through the gustatory system

Our findings demonstrate that FAs are sensed by the primary gustatory system and promote feeding. Flies displayed preference for 6 different FAs tested including hexanoic acid, octanoic acid, decanoic acid, myristic acid, linoleic acid and oleic acid. These represent diverse classes of FAs including short chain and long chain saturated FAs (C6:0 to C14:0) as well as mono- and poly-unsaturated FAs (C18:1, C18:2). These FAs were selected because of known preference by other species of *Drosophila* (short-chain SFAs), preference by *D. melanogaster* larvae and adults (long-chain saturated and unsaturated FAs) or involvement in mosquito's olfactory preference cues (long-chain SFAs) [24], [52], [53]. Flies displayed robust responses to all FAs indicating that they are capable of sensing, and displaying preference for diverse FAs.

Flies with surgically ablated olfactory organs retain robust appetitive response to FAs in CAFE and PER assays, showing that the preference for FAs is fully independent of the olfactory system (Fig. 3B and C). High concentrations of FAs are aversive to flies and inhibit feeding through the gustatory and olfactory systems (Fig. 3C). At high concentrations, the majority of short-chain FAs emits a pungent smell that is repulsive to *Drosophila melanogaster*. Species with unique host-plant preference including *D. sechellia* that feed on ripe *Morinda citrifolia* fruit show preference even to high concentration of short chain FAs [54], suggesting that FA preference/avoidance choice is species-specific and dependent on diet. However, our findings reveal that low concentrations of short chain FAs induce a robust feeding response in *D. melanogaster*, which we demonstrated using two independent gustatory assays (Fig. 2).

We employed the PER assay where only tarsal neurons are stimulated to distinguish between gustatory stimulation and ingestion of FAs. Robust appetitive response to FAs in the tarsal PER assay indicates that post-ingestive feedback is dispensable for detection and preference to FAs (Fig. 2C). Preference for sugars based on nutritional information is sufficient even in the absence of gustatory cues [4]–[6] suggesting that peripheral sensory neurons and internal satiation sensors function independently. It remains to be determined whether flies are capable of sensing FAs through internal metabolic sensors. Future studies examining long-term food choice in *norpA* and *Poxn* mutant flies lacking FA taste may address this question. Fatty acids are hydrophobic chemicals and their texture differs from water or hydrophilic sugar solutions. Flies with genetically silenced gustatory neurons (Gr64f-GAL4>UAS-Kir2.1,GAL80^ts^) do not respond to FAs or sugars (Fig. 4C). Genetic silencing of sugar-sensing neurons does not impair mechanoreceptor function, indicating that the mechanical properties of FAs do not contribute to the FA-induced feeding response. Acid sensing in *Drosophila* regulates egg-laying, food-choice, and avoidance behavior [24],[36],[52],[55]. However, flies robustly respond to HxA buffered to pH∼7 indicating that the appetitive response to FAs is independent of acidity.

In mammals, FAs are detected through mechanosensory, gustatory and olfactory sensory systems [21], [56], [57]. Due to this multi-modal detection, establishing perception of dietary lipids and FAs as a distinct taste modality has been challenging [58], [59]. Previous studies have revealed that *D. melanogaster* can detect FAs, but did not discriminate between feedback from internal satiation sensors, gustatory, or olfactory signals [24], [52]. Our findings demonstrate that FAs are sensed specifically through the gustatory system, independent of acidic properties, mechanical, olfactory, or metabolic feedback. Therefore, in addition to sweet, bitter, salt, water and carbonation, FAs represent a novel taste modality in *Drosophila* [60]–[63].

### Fatty acids signal through sugar-sensing neurons

FAs sensing requires the same neurons that detect sugars and induce feeding behavior. Genetic silencing of Gr64f neurons abolished PER response to all concentrations of HxA and all tested sugars (Fig. 4C). The appetitive response elicited by FA-driven activation of sugar-sensing neurons indicates that these neurons harbor receptors for multiple taste modalities. In addition to sugars and FAs, the same neurons are activated by glycerol, an appetitive and nutritionally relevant alcohol that is detected through the specific receptor Gr64e [64]. The co-expression of multiple appetitive gustatory receptors allows *Drosophila* to categorize food sources in the absence of distinct neurons for each appetitive taste modality. Taken together, these findings support the labeled lines model for gustatory processing, where one subset of sensory neurons confers attractive behavior and the complementary subset confers repulsive behavior [9], [60].

While it is clear that FAs are sensed in gustatory neurons, our findings do not rule out the presence of internal FA receptors. GRs mediating sugar-response are expressed in peripheral sensory neurons, but also in abdominal neurons where they are involved in detection of sugars in hemolymph and in metabolic regulation [25], [65], [66]. Flies can detect and respond to FA-based diet by perception of FAs through their peripheral sensory neurons, but it remains to be determined whether the internal neurons can also perceive FAs and regulate metabolically-relevant processes directly.

### Molecular mechanisms of FA taste

Mutation of the PLC ortholog *norpA* abolishes the appetitive response to FAs, without affecting response to other appetitive taste stimuli including sugars and yeast. Expressing the wild-type allele of *norpA* selectively in sweet-sensing neurons under the control of Gr64f-GAL4 revealed that these neurons are necessary for detection of FAs, and the PLC signaling pathway is selectively required for FAs response. These findings indicate that shared neurons regulate FA and sugar taste, while distinct transduction pathways are involved in processing of each sensation. The *Drosophila* gene *norpA* is an essential component of the transduction pathways in visual and olfactory system [67] and has previously been implicated in TRPA1-dependent taste through function in bitter-sensing neurons [48]. The *Drosophila* genome encodes for two *norpA* isoforms [68]. It is possible that these isoforms have distinct functions that allow for independent regulation of vision and taste. In mice, PLC is selectively expressed in taste cells, and PLC knockout mice do not respond to sweet, amino acid, and bitter tastants [42], [69]. The specific requirement for PLC signaling in FA taste in fly suggests a conserved gustatory transduction pathway that is more similar to mammalian taste than to other taste modalities in *Drosophila*.

PLC-signaling is coupled to diacylgylcerol (DAG) that activates *Drosophila* Transient Receptor Potential (TRP) and TRP-like (TRPL) channels [70], raising the possibility that TRP channels function as FA receptors. dTRPA1 functions in the *Drosophila* brain as a temperature sensor [50] and in the proboscis where it mediates avoidance response in bitter-sensing neurons [48], [49], [71]. In mammals, TRPA1 expresses in taste cells [72] and also functions as a receptor for polyunsaturated fatty acid [47]; however, we find that TRPA1 mutant flies have normal appetitive response to FAs (Fig. S3). In mammals, CD36, a lipid binding protein, is expressed in gustatory oral tissue and appears to be selectively involved in FA taste. CD36 knock-out animals show no preference for FAs but retain their preference for sugars [20],[73]. CD36 is conserved in flies but it is expressed only in olfactory neurons and function in olfactory detection of pheromones that are FA-derived [74]. Future work determining the FA receptors that activate PLC signaling will be central to understanding FA taste in *Drosophila*.

While our findings reveal the importance of PLC signaling in *Drosophila*, we did not identify the receptor(s) required for sensing FAs. A number of GRs have unknown ligands and are co-expressed with Gr5a/Gr64f including Gr61a and Gr61b-d, raising the possibility that these are ligands for FAs [3]. Targeting these receptors selectively in Gr64f-expressing GRNs and testing flies for FA response in the CAFE or PER assays may be useful for identifying the FA receptor. A bioinformatic approach has also been used to search for gustatory receptors in *Drosophila*. Microarray analysis for genes differentially expressed between *Poxn* mutants that lack all chemosensory sensillae and wild-type flies, led to the identification of *pickpocket28*, a *Drosophila* water receptor [63]. We localize FA taste to sweet-sensing neurons and therefore it is feasible to apply cell-sorting techniques followed by expression analysis [75] to reveal candidate receptors signaling FA taste.

### Discrimination of different appetitive substances in *Drosophila*

Previous work demonstrated that *Drosophila* use a relatively simple system of categorizing tastes within a given modality, discriminating distinct sugars based on intensity but not quality [30]. Because FAs are sensed by the same neurons that detect sugars it is possible that flies can only distinguish between FAs and sugars based on concentration-dependent intensity. Alternatively, FAs could be discriminated based on distinct temporal signaling resulting from the different transduction pathway. A parallel system is utilized by bitter-sensing neurons, where certain bitter substances signal through G-protein coupled receptors (GPCRs), and electrophilic tastants signal though TRPA1 channels [49]. Future studies examining FA-conditioned memories may provide insight into gustatory processing in *Drosophila* and advance our understanding of gustatory conditioning. Testing FAs, sugars and glycerol in conditioning discrimination assay [5], [28], [30] may reveal whether different chemical groups are perceived differently based on their chemical structures and underlying transduction pathways.

## Materials and Methods

### Animals

*Drosophila* stocks were maintained on standard cornmeal/agar/molasses medium at 25°C, 70% humidity, in a LD incubator with 12∶12 light/dark cycle. Experiments were performed with wild-type Canton-S flies (From M. Heisenberg, Wuerzburg University) and the following transgenic lines were used: Gr64f-GAL4 (From J. Carlson, Yale University; [76], Kir2.1-GAL4;GAL80*^ts^* (From H. Tanimoto, MPI, Munich; [40]), w;*norpA* ^P24^,UAS-*norpA* (From C. Schnaitmann, MPI, Munich), w;*norpA* ^P24^ [45], w-;;dTrpA1^ins^ [50] .The RNAi lines used to target *norpA* were part of the Transgenic RNAi Project collection from JFRC/HHMI. Bloomington stock #31113 is referred to as *norpA*-IR^#1^ and stock #31197 is referred to as *norpA*-IR^#2^ [77].

### Chemicals

All chemicals used for behavioral assays were purchased from Sigma Aldrich including fructose, sucrose, hexanoic acid, octanoic acid, linoleic acid, acetic acid, oleic acid, decanoic acid, myristic acid, HCl and NaOH. Yeast extract (Bio-Rad, NitroBacter). FAs were first diluted in 80% ethanol in ratio 1∶10, then further diluted in water. Control solutions were also mixed with ethanol to achieve the same final concentration of ethanol. HxA was diluted in PBS buffer to increase pH to 7.2. It was then tested against PBS of pH 7.4. pH was measured by SevenEasy pH Meter, Mettler Toledo, Columbus, OH.

### Behavioral experiments

#### Proboscis extension reflex (PER)

Three to five day old female flies were collected and placed on fresh food for 24 hours, then starved for 24 to 48 hours in food-vials containing wet Kimwipe paper. Only for experiments with *norpA*, males were used for both experimental and control groups. Flies were then anaesthetized under CO_2_, glued with nail polish (Cat#72180, Electron Microscopy Science) on a microscopy slide to their thorax and wings, leaving heads and legs unconstrained [28]. Following 3–6 hours recovery in a humidified chamber the slide was mounted vertically under the dissecting microscope (Leica, S6E) and PER was observed. PER induction was performed as described previously [10]. Briefly, flies were satiated with water before and during experiments. Flies that did not water satiate within 5 mins were excluded from experiments. A 1 ml syringe (Tuberculine, BD&C) with an attached pipette tip (TipOne, # 1111-0200) was used for tastant presentation. Tastant was manually applied to tarsi for 2–3 sec three times with 10 s inter-trial interval and the number of full proboscis extensions was recorded [10], [78]. Tarsi were then washed with distilled water between applications of different tastants and flies were allowed to drink water during the experiment *ad libitum*. Each fly was assayed for response to multiple tastants. PER response was calculated as a percentage of proboscis extensions to total number of tastant stimulations to tarsi [28].

#### Two-choice capillary feeding assay (CAFE)

A modified volumetric drinking assay was used to test food preference [7], [30], [79]. Flies were allowed to drink two solutions presented in capillaries (WPI, #1B150F-4 ID 1 mm, OD 1.5 mm, with filament) attached to an empty food vial and vials were placed in 45° angle. The openings of the capillaries were aligned with the ceiling of the vial. Following 24 or 48 hours of starvation, 30–60 flies were placed into a vial and food consumption was measured. The volume consumed was calculated as the length of liquid missing from the capillary minus the length missing due to evaporation in control capillary tubes, multiplied by the cross-section of the inner diameter of the capillary. Consumption was measured every hour following the introduction of flies into the assay. Taste compounds were mixed with Allura red food dye (FD&C red #40) to a concentration of 3 µl per 1 ml of solution for better visibility in the capillary tube. Following the conclusion of the assay flies were anaesthetized and the number of flies in each vial was counted, corrected for number of flies that died over the course of the experiment (See Survival Index). Total consumption per fly was measured as volume consumed in each capillary divided by number of live flies in the vial. Preference Index (PI) was calculated as volume consumed from capillary with test solution minus volume consumed from capillary with control solution, divided by total volume consumed.

#### Survival index

Flies were starved for 48 hours prior to the experiment. To calculate survival index, the number of dead flies in CAFE vials was counted over the time starting at 3 minutes after onset of the experiment until 24 hours. At the end of the experiment, all flies were counted and the ratio of survivals was calculated.

### Statistics

Statistical analyses were performed using InStat software (GraphPad Software 5.0 Inc.). For PER experiments, most tested groups violated the assumption of the normal distribution. Therefore, all the data were analyzed with non-parametric statistics. All experiments include data from >20 flies. Each fly was sampled three times with the same stimulus. The response was binary (PER yes/no), and these three responses were pooled for values ranging from 0 to 3. Kruskal-Wallis test (nonparametric ANOVA) was performed on the raw data from single flies and Dunn's Multiple Comparisons test was used to compare different groups. For capillary feeding assay, 30–60 flies were used per tube and 4 to 20 tubes per group were tested. Wilcoxon signed rank test (non-parametric) with two-tailed P value was used to test significance on single groups. In figures, graph bars are mean values and error bars are standard error of the mean.

## Supporting Information

Figure S1 Survival dose-response curve for hexanoic acid. Flies were starved for 48 hrs prior to the start of the experiment, and survival was measured for 24 hrs while flies were fed a diet composed of 1%, 0.4% or 0.01% hexanoic acid (HxA). Flies with access to water alone had significantly reduced survival rate compared to HxA fed flies. All data, mean ± s.e.m. ** p<0.01, *** p<0.001; NS, not significant.
(PDF)

Figure S2 Appetitive response to HxA in two-choice CAFE assay is comparable to low concentrations of fructose and is concentration dependent. Intake of 0.4% HxA was measured against different concentrations of fructose. Flies prefer 0.4% HxA to 1 mM fructose or lower, while fructose is preferred at concentrations of 2 mM and greater (p<0.001). All data, mean ± s.e.m. ** p<0.01, *** p<0.001; NS, not significant, t-test.
(TIF)

Figure S3 Fatty acid taste detection requires *norpA* in sweet-sensing neurons and is independent of *norpA* expression in the eye and bitter-sensing neurons, and of *TRPA1*. PER response was measured in dTrpA1^ins^ and *norpA* ^P24^ rescue flies. Expression of *norpA* limited to the *rhopdopisin-1* expressing neurons or bitter-sensing Gr66a-neurons in *norpA* mutant background, does not rescue response to HxA. TRPA1 mutant flies (dTrpA1^ins^) display wild-type response to HxA. All data, mean ± s.e.m. ** p<0.01, *** p<0.001; NS, not significant, t-test.
(TIF)

Figure S4 Targeted knockdown of *norpA* significantly reduces PER to HxA. PER response to 0.4% HxA, 10 mM and 100 mM fructose, and 1 M sucrose was measured in flies expressing *norpA* RNAi in Gr64f-expressing neurons. Two RNAi constructs marked IR1 (#31197) and IR2 (#31113) both significantly reduce response to 0.4% HxA compared to control parental lines. Response to sugars remains the same in flies with blocked *norpA* as compared to control lines; except a small decrease of response to 10 mM fructose in Gr64f>norpA-IR2. . All data, mean ± s.e.m. * p<0.05, ** p<0.01, *** p<0.001; NS, not significant.
(TIF)

## References

[1] Scott K (2005) Taste recognition: food for thought. Neuron 48: 455–464. 16269362 10.1016/j.neuron.2005.10.015

[2] Yarmolinsky Da, Zuker CS, Ryba NJP (2009) Common sense about taste: from mammals to insects. Cell 139: 234–244. 19837029 10.1016/j.cell.2009.10.001 PMC3936514

[3] Montell C (2009) A taste of the Drosophila gustatory receptors. Current opinion in neurobiology 19: 345–353. 19660932 10.1016/j.conb.2009.07.001 PMC2747619

[4] Fujita M, Tanimura T (2011) Drosophila Evaluates and Learns the Nutritional Value of Sugars. Current Biology 21: 751–755. 21514154 10.1016/j.cub.2011.03.058

[5] Burke CJ, Waddell S (2011) Report Remembering Nutrient Quality of Sugar in Drosophila. Current Biology 21: 746–750. 21514159 10.1016/j.cub.2011.03.032 PMC3094154

[6] Dus M, Min S, Keene AC, Young G, Suh GSB (2011) Taste-independent detection of the caloric content of sugar in Drosophila. Direct 2–7. 10.1073/pnas.1017096108 PMC3136275 21709242

[7] Stafford JW, Lynd KM, Jung AY, Gordon MD (2012) Integration of taste and calorie sensing in Drosophila. J Neurosci 32: 14767–14774. 23077061 10.1523/JNEUROSCI.1887-12.2012 PMC6621435

[8] Marella S, Fischler W, Kong P, Asgarian S, Rueckert E, et al (2006) Imaging taste responses in the fly brain reveals a functional map of taste category and behavior. Neuron 49: 285–295. 16423701 10.1016/j.neuron.2005.11.037

[9] Amrein H, Thorne N (2005) Gustatory Perception and Behavior in Drosophila melanogaster. Current 15: 673–684. 10.1016/j.cub.2005.08.021 16139201

[10] Wang Z, Singhvi A, Kong P, Scott K (2004) Taste representations in the Drosophila brain. Cell 117: 981–991. 15210117 10.1016/j.cell.2004.06.011

[11] Thorne N, Chromey C, Bray S, Amrein H, Building C, et al (2004) Taste Perception and Coding in Drosophila. Current 14: 1065–1079. 10.1016/j.cub.2004.05.019 15202999

[12] Tepper BJ, Nurse RJ (1997) Fat perception is related to PROP taster status. Physiol Behav 61: 949–954. 9177570 10.1016/s0031-9384(96)00608-7

[13] Ramirez I (1994) Chemosensory similarities among oils: does viscosity play a role? Chem Senses 19: 155–168. 8055265 10.1093/chemse/19.2.155

[14] Kinney NE, Antill RW (1996) Role of olfaction in the formation of preference for high-fat foods in mice. Physiol Behav 59: 475–478. 8700949 10.1016/0031-9384(95)02086-1

[15] Greenberg D, Smith GP (1996) The controls of fat intake. Psychosom Med 58: 559–569. 8948004 10.1097/00006842-199611000-00004

[16] Takeda M, Sawano S, Imaizumi M, Fushiki T (2001) Preference for corn oil in olfactory-blocked mice in the conditioned place preference test and the two-bottle choice test. Life Sci 69: 847–854. 11487096 10.1016/s0024-3205(01)01180-8

[17] Fukuwatari T, Shibata K, Iguchi K, Saeki T, Iwata A, et al (2003) Role of gustation in the recognition of oleate and triolein in anosmic rats. Physiol Behav 78: 579–583. 12782211 10.1016/s0031-9384(03)00037-4

[18] Hiraoka T, Fukuwatari T, Imaizumi M, Fushiki T (2003) Effects of oral stimulation with fats on the cephalic phase of pancreatic enzyme secretion in esophagostomized rats. Physiol Behav 79: 713–717. 12954413 10.1016/s0031-9384(03)00201-4

[19] Kawai T, Fushiki T (2003) Importance of lipolysis in oral cavity for orosensory detection of fat. Am J Physiol Regul Integr Comp Physiol 285: R447–454. 12702486 10.1152/ajpregu.00729.2002

[20] Laugerette F, Passilly-Degrace P, Patris B, Niot I, Febbraio M, et al (2005) CD36 involvement in orosensory detection of dietary lipids, spontaneous fat preference, and digestive secretions. J Clin Invest 115: 3177–3184. 16276419 10.1172/JCI25299 PMC1265871

[21] Chale-Rush A, Burgess JR, Mattes RD (2007) Multiple routes of chemosensitivity to free fatty acids in humans. Am J Physiol Gastrointest Liver Physiol 292: G1206–1212. 17234892 10.1152/ajpgi.00471.2006

[22] Cartoni C, Yasumatsu K, Ohkuri T, Shigemura N, Yoshida R, et al (2010) Taste preference for fatty acids is mediated by GPR40 and GPR120. J Neurosci 30: 8376–8382. 20573884 10.1523/JNEUROSCI.0496-10.2010 PMC6634626

[23] Smith JC (2010) Orosensory Factors in Fat Detection. In: Montmayeur JP, le Coutre J, editors. Fat Detection: Taste, Texture, and Post Ingestive Effects. Boca Raton (FL). 21452472

[24] Harada E, Haba D, Aigaki T, Matsuo T (2008) Behavioral analyses of mutants for two odorant-binding protein genes, Obp57d and Obp57e, in Drosophila melanogaster. Genes Genet Syst 83: 257–264. 18670137 10.1266/ggs.83.257

[25] Thorne N, Amrein H (2008) Atypical Expression of Drosophila Gustatory Receptor Genes in Sensory. Receptor 568: 548–568. 10.1002/cne.21547 18067151

[26] Marella S, Mann K, Scott K (2012) Dopaminergic modulation of sucrose acceptance behavior in Drosophila. Neuron 73: 941–950. 22405204 10.1016/j.neuron.2011.12.032 PMC3310174

[27] Shiraiwa T, Carlson JR (2007) Proboscis extension response (PER) assay in Drosophila. J Vis Exp 193. 18978998 10.3791/193 PMC2535836

[28] Keene AC, Masek P (2012) Optogenetic induction of aversive taste memory. Neuroscience 222: 173–180. 22820051 10.1016/j.neuroscience.2012.07.028 PMC4006090

[29] Awasaki T, Kimura K (1997) pox-neuro is required for development of chemosensory bristles in Drosophila. J Neurobiol 32: 707–721. 9183748 10.1002/(sici)1097-4695(19970620)32:7<707::aid-neu6>3.0.co;2-8

[30] Masek P, Scott K (2010) Limited taste discrimination in Drosophila. Proceedings of the National Academy of Sciences of the United States of America 107: 14833–14838. 20679196 10.1073/pnas.1009318107 PMC2930483

[31] Clyne PJ, Warr CG, Carlson JR (2000) Candidate taste receptors in Drosophila. Science 287: 1830–1834. 10710312 10.1126/science.287.5459.1830

[32] Robertson HM, Warr CG, Carlson JR (2003) Molecular evolution of the insect chemoreceptor gene superfamily in Drosophila melanogaster. Proc Natl Acad Sci U S A 100 Suppl 2: 14537–14542. 14608037 10.1073/pnas.2335847100 PMC304115

[33] Gerber B, Stocker RF, Tanimura T, Thum AS (2008) Smelling , Tasting , Learning : Drosophila as a Study Case. 1–47. 10.1007/400_2008_9 19145411

[34] Shiraiwa T (2008) Multimodal chemosensory integration through the maxillary palp in Drosophila. PloS one 3: e2191. 18478104 10.1371/journal.pone.0002191 PMC2364657

[35] Charro MJ, Alcorta E (1994) Quantifying relative importance of maxillary palp information on the olfactory behavior of Drosophila melanogaster. J Comp Physiol A 175: 761–766. 7807417 10.1007/BF00191847

[36] Ai M, Min S, Grosjean Y, Leblanc C, Bell R, et al (2010) Acid sensing by the Drosophila olfactory system. Nature 468: 691–695. 21085119 10.1038/nature09537 PMC3105465

[37] Slone J, Daniels J, Amrein H (2007) Sugar receptors in Drosophila. Curr Biol 17: 1809–1816. 17919910 10.1016/j.cub.2007.09.027 PMC2078200

[38] Jiao Y, Moon SJ, Wang X, Ren Q, Montell C (2008) Gr64f is required in combination with other gustatory receptors for sugar detection in Drosophila. Curr Biol 18: 1797–1801. 19026541 10.1016/j.cub.2008.10.009 PMC2676565

[39] Baines RA, Uhler JP, Thompson A, Sweeney ST, Bate M (2001) Altered electrical properties in Drosophila neurons developing without synaptic transmission. J Neurosci 21: 1523–1531. 11222642 10.1523/JNEUROSCI.21-05-01523.2001 PMC6762927

[40] Thum AS, Knapek S, Rister J, Dierichs-schmitt EVA, Heisenberg M, et al (2006) Differential Potencies of Effector Genes in Adult Drosophila. Time 203: 194–203. 10.1002/cne.21022 16856137

[41] McGuire SE, Le PT, Osborn AJ, Matsumoto K, Davis RL (2003) Spatiotemporal rescue of memory dysfunction in Drosophila. Science 302: 1765–1768. 14657498 10.1126/science.1089035

[42] Zhang Y, Hoon MA, Chandrashekar J, Mueller KL, Cook B, et al (2003) Coding of Sweet , Bitter , and Umami Tastes : Different Receptor Cells Sharing Similar Signaling Pathways. Receptor 112: 293–301. 10.1016/s0092-8674(03)00071-0 12581520

[43] Rossler P, Kroner C, Freitag J, Noe J, Breer H (1998) Identification of a phospholipase C beta subtype in rat taste cells. Eur J Cell Biol 77: 253–261. 9860142 10.1016/s0171-9335(98)80114-3

[44] Yoshida Y, Saitoh K, Aihara Y, Okada S, Misaka T, et al (2007) Transient receptor potential channel M5 and phospholipaseC-beta2 colocalizing in zebrafish taste receptor cells. Neuroreport 18: 1517–1520. 17885593 10.1097/WNR.0b013e3282ec6874

[45] Hardie RC, Martin F, Chyb S, Raghu P (2003) Rescue of light responses in the Drosophila “null” phospholipase C mutant, norpAP24, by the diacylglycerol kinase mutant, rdgA, and by metabolic inhibition. J Biol Chem 278: 18851–18858. 12621055 10.1074/jbc.M300310200

[46] Chyb S, Raghu P, Hardie RC (1999) Polyunsaturated fatty acids activate the Drosophila light-sensitive channels TRP and TRPL. Nature 397: 255–259. 9930700 10.1038/16703

[47] Motter AL, Ahern GP (2012) TRPA1 is a polyunsaturated fatty acid sensor in mammals. PLoS One 7: e38439. 22723860 10.1371/journal.pone.0038439 PMC3378573

[48] Kim SH, Lee Y, Akitake B, Woodward OM, Guggino WB, et al (2010) Drosophila TRPA1 channel mediates chemical avoidance in gustatory receptor neurons. Proc Natl Acad Sci U S A 107: 8440–8445. 20404155 10.1073/pnas.1001425107 PMC2889570

[49] Kang K, Panzano VC, Chang EC, Ni L, Dainis AM, et al (2012) Modulation of TRPA1 thermal sensitivity enables sensory discrimination in Drosophila. Nature 481: 76–80. 22139422 10.1038/nature10715 PMC3272886

[50] Hamada FN, Rosenzweig M, Kang K, Pulver SR, Ghezzi A, et al (2008) An internal thermal sensor controlling temperature preference in Drosophila. Nature 454: 217–220. 18548007 10.1038/nature07001 PMC2730888

[51] Wisotsky Z, Medina A, Freeman E, Dahanukar A (2011) Evolutionary differences in food preference rely on Gr64e, a receptor for glycerol. Nature neuroscience 14: 1534–1541. 22057190 10.1038/nn.2944

[52] Fougeron A-s, Farine J-p, Flaven-pouchon J, Everaerts C (2011) Fatty-Acid Preference Changes during Development in Drosophila melanogaster. October 6. 10.1371/journal.pone.0026899 PMC3203165 22046401

[53] Bosch OJ, Geier M, Boeckh J (2000) Contribution of fatty acids to olfactory host finding of female Aedes aegypti. Chem Senses 25: 323–330. 10866990 10.1093/oxfordjournals.chemse.a014042

[54] Matsuo T, Sugaya S, Yasukawa J, Aigaki T, Fuyama Y (2007) Odorant-binding proteins OBP57d and OBP57e affect taste perception and host-plant preference in Drosophila sechellia. PLoS Biol 5: e118. 17456006 10.1371/journal.pbio.0050118 PMC1854911

[55] Joseph RM, Devineni AV, King IFG, Heberlein U (2009) Oviposition preference for and positional avoidance of acetic acid provide a model for competing behavioral drives in Drosophila. Proceedings of the National Academy of Sciences of the United States of America 106: 11352–11357. 19541615 10.1073/pnas.0901419106 PMC2698888

[56] Gilbertson TA, Liu L, Kim I, Burks CA, Hansen DR (2005) Fatty acid responses in taste cells from obesity-prone and -resistant rats. Physiol Behav 86: 681–690. 16249010 10.1016/j.physbeh.2005.08.057

[57] Pittman DW, Labban CE, Anderson AA, O'Connor HE (2006) Linoleic and oleic acids alter the licking responses to sweet, salt, sour, and bitter tastants in rats. Chem Senses 31: 835–843. 16923777 10.1093/chemse/bjl026

[58] Mattes RD (2010) Fat Taste in Humans: Is It a Primary? In: Montmayeur JP, le Coutre J, editors. Fat Detection: Taste, Texture, and Post Ingestive Effects. Boca Raton (FL).

[59] Tucker RM, Mattes RD (2012) Are free fatty acids effective taste stimuli in humans? Presented at the symposium “The Taste for Fat: New Discoveries on the Role of Fat in Sensory Perception, Metabolism, Sensory Pleasure and Beyond” held at the iNstitute of Food Technologists 2011 Annual Meeting, New Orleans, LA, June 12, 2011. J Food Sci 77: S148–151. 22384969 10.1111/j.1750-3841.2011.02518.x

[60] Marella S, Fischler W, Kong P, Asgarian S, Rueckert E, et al (2006) Imaging taste responses in the fly brain reveals a functional map of taste category and behavior. Neuron 49: 285–295. 16423701 10.1016/j.neuron.2005.11.037

[61] Liu L, Leonard AS, Motto DG, Feller MA, Price MP, et al (2003) Contribution of Drosophila DEG/ENaC genes to salt taste. Neuron 39: 133–146. 12848938 10.1016/s0896-6273(03)00394-5

[62] Fischler W, Kong P, Marella S, Scott K (2007) The detection of carbonation by the Drosophila gustatory system. Nature 448: 1054–1057. 17728758 10.1038/nature06101

[63] Cameron P, Hiroi M, Ngai J, Scott K (2010) The molecular basis for water taste in Drosophila. Nature 465: 91–95. 20364123 10.1038/nature09011 PMC2865571

[64] Wisotsky Z, Medina A, Freeman E, Dahanukar A (2011) Evolutionary differences in food preference rely on Gr64e, a receptor for glycerol. Nat Neurosci 14: 1534–1541. 22057190 10.1038/nn.2944

[65] Stocker RF (1994) The organization of the chemosensory system in Drosophila melanogaster: a review. Cell Tissue Res 275: 3–26. 8118845 10.1007/BF00305372

[66] Ikeya T, Galic M, Belawat P, Nairz K, Hafen E (2002) Nutrient-dependent expression of insulin-like peptides from neuroendocrine cells in the CNS contributes to growth regulation in Drosophila. Curr Biol 12: 1293–1300. 12176357 10.1016/s0960-9822(02)01043-6

[67] Bloomquist BT, Shortridge RD, Schneuwly S, Perdew M, Montell C, et al (1988) Isolation of a putative phospholipase C gene of Drosophila, norpA, and its role in phototransduction. Cell 54: 723–733. 2457447 10.1016/s0092-8674(88)80017-5

[68] Kim S, McKay RR, Miller K, Shortridge RD (1995) Multiple subtypes of phospholipase C are encoded by the norpA gene of Drosophila melanogaster. J Biol Chem 270: 14376–14382. 7540168 10.1074/jbc.270.24.14376

[69] Yan W, Sunavala G, Rosenzweig S, Dasso M, Brand JG, et al (2001) Bitter taste transduced by PLC-beta(2)-dependent rise in IP(3) and alpha-gustducin-dependent fall in cyclic nucleotides. Am J Physiol Cell Physiol 280: C742–751. 11245589 10.1152/ajpcell.2001.280.4.C742

[70] Hardie RC (2007) TRP channels and lipids: from Drosophila to mammalian physiology. J Physiol 578: 9–24. 16990401 10.1113/jphysiol.2006.118372 PMC2075119

[71] Al-Anzi B, Tracey WD Jr, Benzer S (2006) Response of Drosophila to wasabi is mediated by painless, the fly homolog of mammalian TRPA1/ANKTM1. Curr Biol 16: 1034–1040. 16647259 10.1016/j.cub.2006.04.002

[72] Perez CA, Huang L, Rong M, Kozak JA, Preuss AK, et al (2002) A transient receptor potential channel expressed in taste receptor cells. Nat Neurosci 5: 1169–1176. 12368808 10.1038/nn952

[73] Sclafani A, Ackroff K, Abumrad NA (2007) CD36 gene deletion reduces fat preference and intake but not post-oral fat conditioning in mice. Am J Physiol Regul Integr Comp Physiol 293: R1823–1832. 17804586 10.1152/ajpregu.00211.2007

[74] Benton R, Vannice KS, Vosshall LB (2007) An essential role for a CD36-related receptor in pheromone detection in Drosophila. Nature 450: 289–293. 17943085 10.1038/nature06328

[75] Nagoshi E, Sugino K, Kula E, Okazaki E, Tachibana T, et al (2010) Dissecting differential gene expression within the circadian neuronal circuit of Drosophila. Nat Neurosci 13: 60–68. 19966839 10.1038/nn.2451 PMC3878269

[76] Dahanukar A, Lei Y-t, Kwon JY, Carlson JR (2007) Article Two Gr Genes Underlie Sugar Reception in Drosophila. Neuron 503–516. 17988633 10.1016/j.neuron.2007.10.024 PMC2096712

[77] Ni JQ, Markstein M, Binari R, Pfeiffer B, Liu LP, et al (2008) Vector and parameters for targeted transgenic RNA interference in Drosophila melanogaster. Nat Methods 5: 49–51. 18084299 10.1038/nmeth1146 PMC2290002

[78] Chabaud MA, Devaud JM, Pham-Delegue MH, Preat T, Kaiser L (2006) Olfactory conditioning of proboscis activity in Drosophila melanogaster. J Comp Physiol A Neuroethol Sens Neural Behav Physiol 192: 1335–1348. 16964495 10.1007/s00359-006-0160-3

[79] Ja WW, Carvalho GB, Mak EM, de la Rosa NN, Fang AY, et al (2007) Prandiology of Drosophila and the CAFE assay. Proc Natl Acad Sci U S A 104: 8253–8256. 17494737 10.1073/pnas.0702726104 PMC1899109

