# Supplementary figures and images for: Drosophila Fatty Acid Taste Signals through the PLC Pathway in Sugar-Sensing Neurons
Source: PLoS Genet. 2013 Sep 12;9(9):e1003710. doi: 10.1371/journal.pgen.1003710 (PMC3772025; doi:10.1371/journal.pgen.1003710)

Supplementary Fig .1

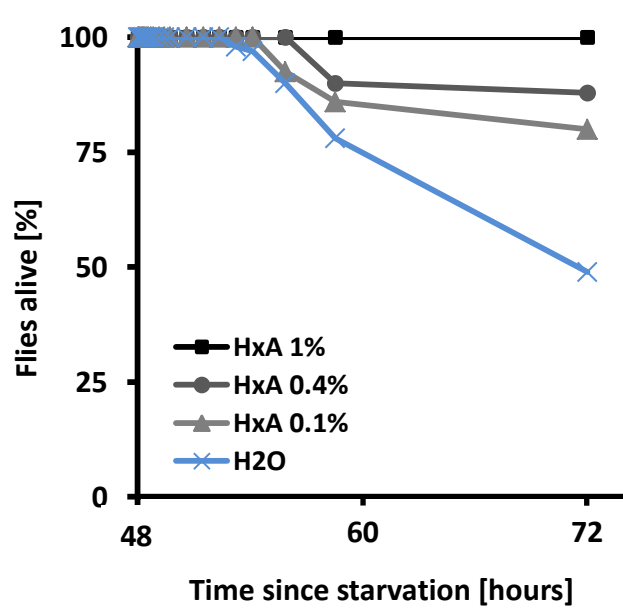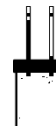

Supplement: Figure S1 — Survival dose-response curve for hexanoic acid. Flies were starved for 48 hrs prior to the start of the experiment, and survival was measured for 24 hrs while flies were fed a diet composed of 1%, 0.4% or 0.01% hexanoic acid (HxA). Flies with access to water alone had significantly reduced survival rate compared to HxA fed flies. All data, mean ± s.e.m. ** p<0.01, *** p<0.001; NS, not significant. (PDF) [file pgen.1003710.s001.pdf]

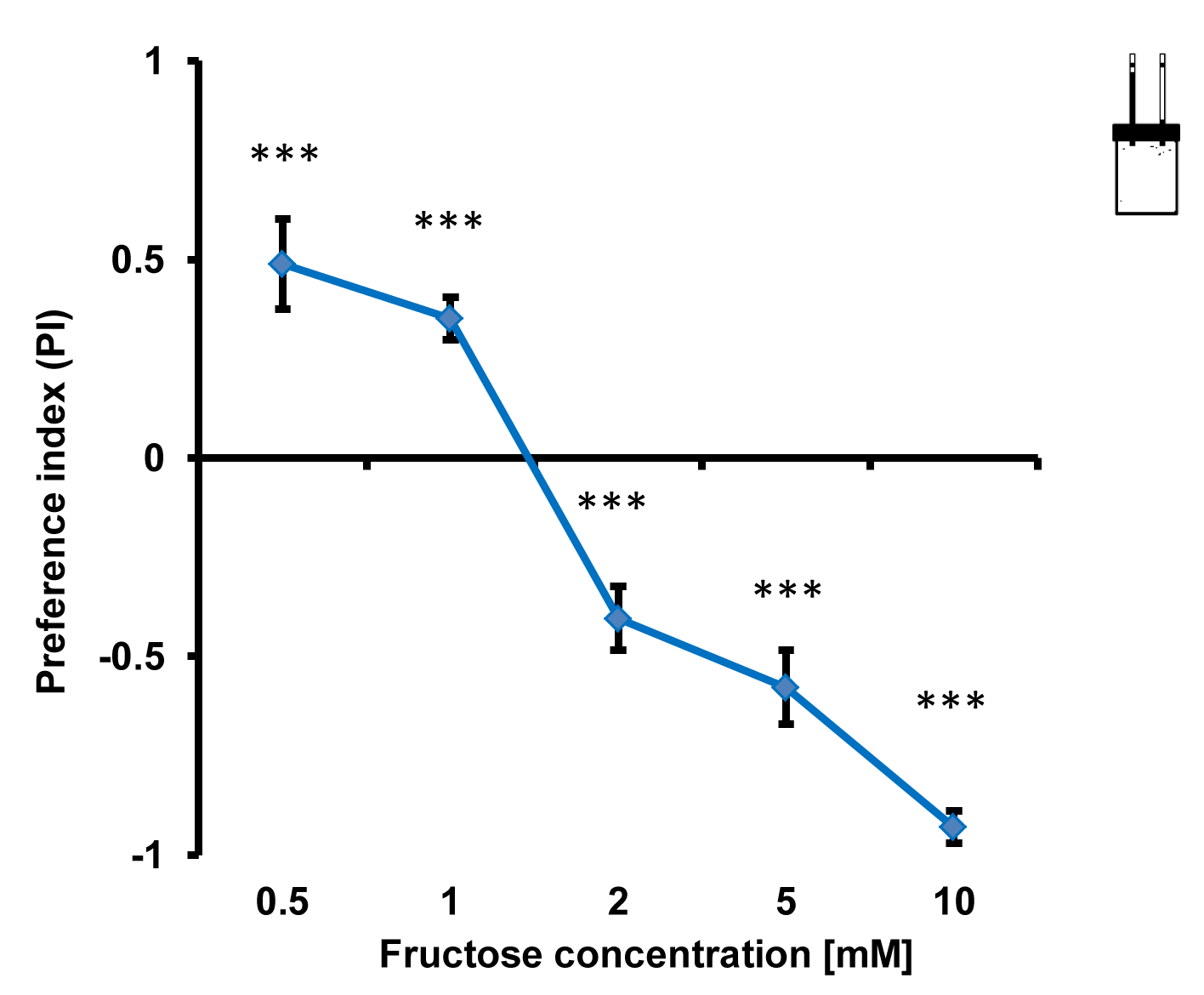

Supplement: Figure S2 — Appetitive response to HxA in two-choice CAFE assay is comparable to low concentrations of fructose and is concentration dependent. Intake of 0.4% HxA was measured against different concentrations of fructose. Flies prefer 0.4% HxA to 1 mM fructose or lower, while fructose is preferred at concentrations of 2 mM and greater (p<0.001). All data, mean ± s.e.m. ** p<0.01, *** p<0.001; NS, not significant, t-test. (TIF) [file pgen.1003710.s002.tif]

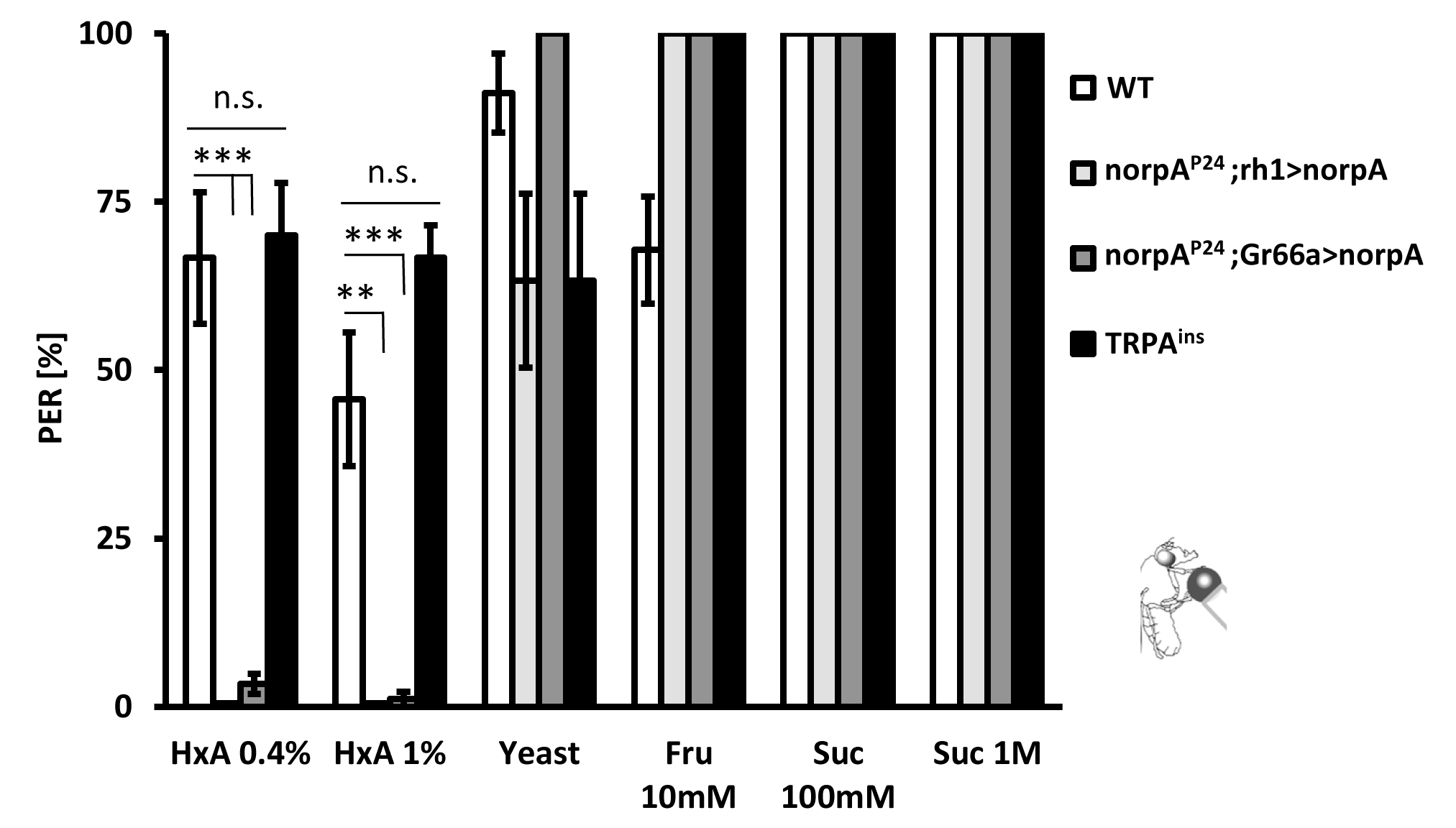

Supplement: Figure S3 — Fatty acid taste detection requires norpA in sweet-sensing neurons and is independent of norpA expression in the eye and bitter-sensing neurons, and of TRPA1. PER response was measured in dTrpA1ins and norpA P24 rescue flies. Expression of norpA limited to the rhopdopisin-1 expressing neurons or bitter-sensing Gr66a-neurons in norpA mutant background, does not rescue response to HxA. TRPA1 mutant flies (dTrpA1ins) display wild-type response to HxA. All data, mean ± s.e.m. ** p<0.01, *** p<0.001; NS, not significant, t-test. (TIF) [file pgen.1003710.s003.tif]

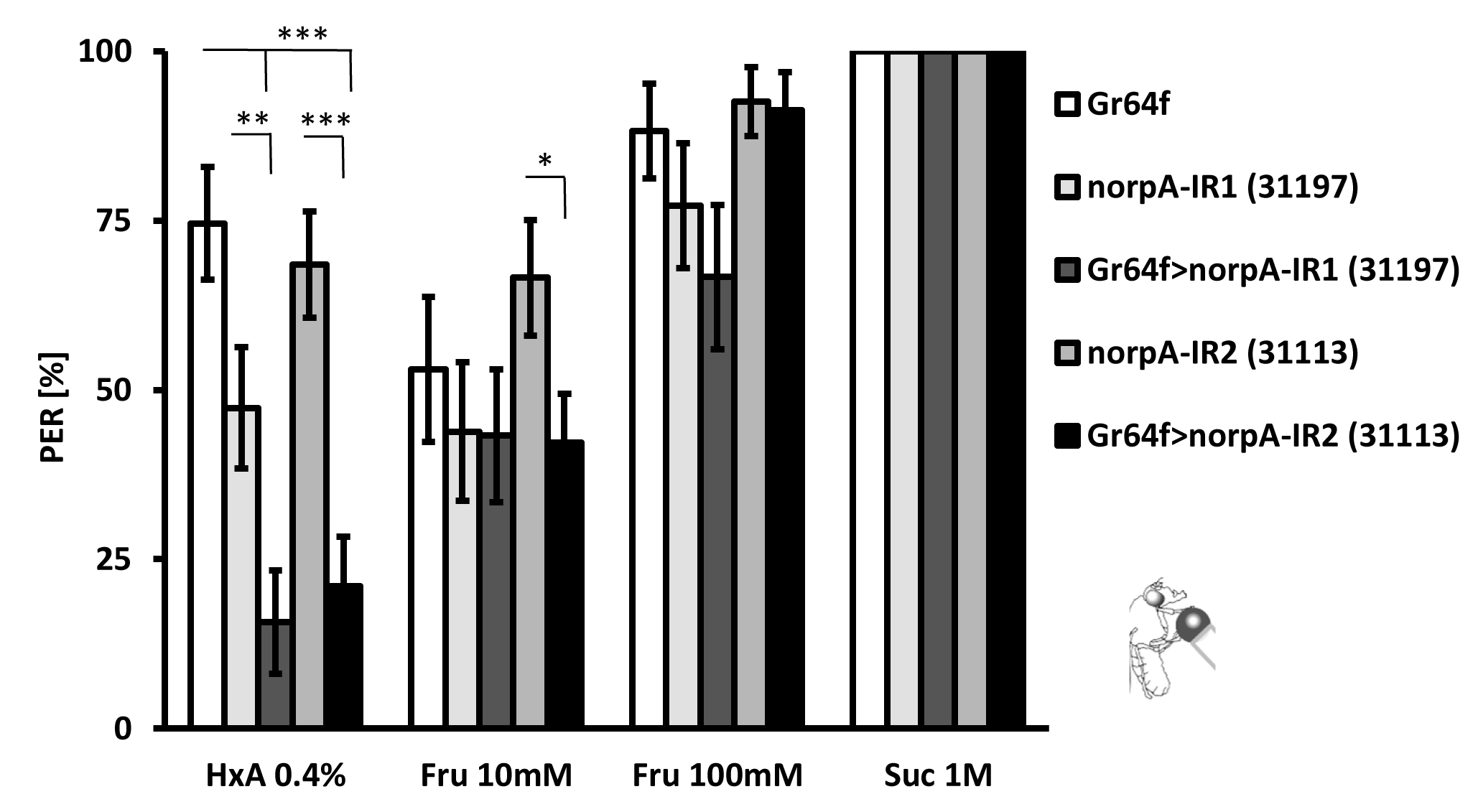

Supplement: Figure S4 — Targeted knockdown of norpA significantly reduces PER to HxA. PER response to 0.4% HxA, 10 mM and 100 mM fructose, and 1 M sucrose was measured in flies expressing norpA RNAi in Gr64f-expressing neurons. Two RNAi constructs marked IR1 (#31197) and IR2 (#31113) both significantly reduce response to 0.4% HxA compared to control parental lines. Response to sugars remains the same in flies with blocked norpA as compared to control lines; except a small decrease of response to 10 mM fructose in Gr64f>norpA-IR2. . All data, mean ± s.e.m. * p<0.05, ** p<0.01, *** p<0.001; NS, not significant. (TIF) [file pgen.1003710.s004.tif]
